# Ganglion cells and displaced amacrine cells density in the retina of the collared peccary (*Pecari tajacu*)

**DOI:** 10.1371/journal.pone.0239719

**Published:** 2020-10-01

**Authors:** Kelly Helorany Alves Costa, Bruno Duarte Gomes, Luiz Carlos de Lima Silveira, Givago da Silva Souza, Isabelle Christine Vieira da Silva Martins, Eliza Maria da Costa Brito Lacerda, Fernando Allan de Farias Rocha

**Affiliations:** 1 Instituto de Ciências Biológicas, Universidade Federal do Pará, Belém, Pará, Brasil; 2 Núcleo de Medicina Tropical, Universidade Federal do Pará, Belém, Pará, Brasil; 3 Universidade CEUMA, São Luís, Maranhão, Brasil; National Eye Centre, UNITED STATES

## Abstract

In the present study, we investigated the topographical distribution of ganglion cells and displaced amacrine cells in the retina of the collared peccary (*Pecari tajacu*), a diurnal neotropical mammal of the suborder Suina (Order Artiodactyla) widely distributed across central and mainly South America. Retinas were prepared and processed following the Nissl staining method. The number and distribution of retinal ganglion cells and displaced amacrine cells were determined in six flat-mounted retinas from three animals. The average density of ganglion cells was 351.822 ± 31.434 GC/mm^2^. The peccary shows a well-developed visual streak. The average peak density was 6,767 GC/mm^2^ and located within the visual range and displaced temporally as an area temporalis. Displaced amacrine cells have an average density of 300 DAC/mm^2^, but the density was not homogeneous along the retina, closer to the center of the retina the number of cells decreases and when approaching the periphery the density increases, in addition, amacrine cells do not form retinal specialization like ganglion cells. Outside the area temporalis, amacrine cells reach up to 80% in the ganglion cell layer. However, in the region of the area temporalis, the proportion of amacrine cells drops to 32%. Thus, three retinal specializations were found in peccary’s retina by ganglion cells: visual streak, *area temporalis* and dorsotemporal extension. The topography of the ganglion cells layer in the retina of the peccary resembles other species of Order Artiodactyla already described and is directly related to its evolutionary history and ecology of the species.

## Introduction

The Amazon rainforest is the most biodiverse biome of the planet. It is the home of many animal species, including mammals, thus being a significant source of data for comparative anatomy and physiology of tropical wildlife. Throughout the years, several studies have focused on the visual system morphophysiological organization in rodents and primates [1–22].

The Order Artiodactyla has been the target of many studies that aimed to characterize the morphology and physiology of retinal cells. Methods that used morphological and electrophysiological analysis have shown a dichromatic vision supported by short and medium-wavelength sensitive cone cells [23–29]. More specifically, in the ganglion cells layer, the topography distribution was studied in species such as the domestic pig–*Sus scrofa* [30, 31], the giraffe—*Giraffa camelopardalis* [32], Hippopotamus—*Hippopotamus amphibius* [33] goat—*Murciano-granadina breed* [34], and the sheep—*Ovis aries* [35]. These species presented similar topographical ganglion cells distribution: the presence of a high cellular density region elongated horizontally and situated above the optic disc, known as visual streak; a density peak along the visual streak that is temporally dislocated and known as *area temporalis* [36]. This spatial variation of the cell density was also observed for the photoreceptors cone type with short and medium wavelengths in the retina of pigs [29].

The collared peccary (*Pecari tajacu*) is a neotropical mammal of the suborder Suina (Order Artiodactyla), morphologically similar to the suidae of the Old World [37] The collared peccary is widely distributed across central and mainly South America. They have a diurnal/crepuscular activity, feeding in the early to mid-morning and late afternoon to the early hours of the night [38]. For peccary, Ahnelt et al., pointed out that peccaries and suits have similar photoreceptor morphology with rods and cones area easily distinguishable [28]. Furthermore, cones had a high concentration in temporal retina of which the S cones, were arranged in a random mosaic and comprised 10% of the total cone cell population as it is observed for that type of cone in other mammals [28]. Recently, Ezra-Alia et al., have reported more detailed aspects of the peccary retina. They showed that the amount of M/L cones is greater than S cones, and that the amplitude of the combined response of cones and rods is smaller compared to domestic pigs, but very similar to minipigs [39].

The characterization of the ganglion cell distribution and density is still missing for the collared peccary. Thus, in the present study, we aimed to fill this gap using whole-mounted retinas and Nissl staining to investigate the ganglion cell topography of the collared peccary.

## Materials and methods

### Ethical aspects

Experiments were performed with three adults collared peccary (*P*. *tacaju*), all males. With the weight between 15–22 kg and age about 2.5 years (±0.6). The animals were obtained from Empresa Brasileira de Pesquisa Agropecuária—Embrapa/Pará. The maintenance and handling procedures were reviewed and approved by the research ethics committee of the Universidade Federal do Pará (CEPAE, N° 034–2015).

### Animals

The peccaries were euthanized with a 50 mg/kg lethal intraperitoneal injection of sodium thiopental (Thionembutal, Abbott, São Paulo, Brazil) and later the eyes were removed for the present research. The eyes were enucleated immediately after death, cornea and lens were removed. Eyecups were fixed by immersion in 10% formaldehyde in 0.1 M phosphate buffer, pH 7.4. After fixation, whole mounts of the retina were prepared and processed following the Nissl staining method [15, 32, 40]. For retinal orientation first the optic nerve was identified by its conspicuous oval appearance and temporal displacement. Next, five cuts were performed as follow, one cut at each nasal and temporal ends just below the optic nerve, one cut at the ventral end and two cuts in the ends of the diagonal direction.

For the technique described above only six retinas from three animals (all male) were used, firmly adhered to vitreal side up in gelatinized slice, was incubated in formaldehyde vapors for two hours at 60 ºC. Next, the retina was washed in distilled water and stained with 0.5% cresyl violet for 10 min and dehydrated in a series of graded ethanol concentrations, cleared in xylene and coverslipped with Permount.

### Imaging and analysis

The ganglion and amacrine cells were viewed and digitally documented using a microscope Axion Scope A1 (Carl Zeiss) with camera Axiocam HRc. The cell counting was made directly in the microscope using square fields and an objective A-plan 100x/1.25 Oil (Carl Zeiss). The cells were counting at 1 mm intervals along two meridians: (1) horizontal, in the naso-temporal axis along the visual streak; (2) vertical, dorsal-ventral axis, which crosses the horizontal meridian and the optic disc perpendicularly. Counts were also made along the vertical meridian from temporal to nasal border. For some special-target areas, as the *visual streak* or *area temporalis*, the interval between the counting fields was 0.5 mm and 0.25 mm, respectively.

The ganglion and amacrine cell counts were converted to density in cells/mm^2^, and the estimation of the isodensity lines was described in earlier studies of our group [15, 21]. Briefly, the isodensity contours were plotted linking the points over an isodensity contour and the points located between densities higher and lower than that corresponding to the isodensity contours. The total number of ganglion cells (GC) and displaced amacrine cells were then obtained by a simple measure of the area between two isodensity contours multiplying the area times the mean density value of the two isodensity contours. The total number was then the sum of all the resulting isodensity figures. To color the topographic maps the isodensity contours were drawn and converted into color-coded isodensity lines using SigmaPlot^®^ for Windows™ Version 12.5 software (Systat Software, Inc., Richmond, CA).

### Statistical analysis

For descriptive statistics and plotting of Figs 5, 6, 7 and 9, we used the SigmaStat^®^ for Windows™ Version 3.11 program (Systat Software, Inc., Richmond, CA).

## Results

### Gross anatomy, retinal area and identification of ganglion cells and displaced amacrine cells

**Fig 1:**
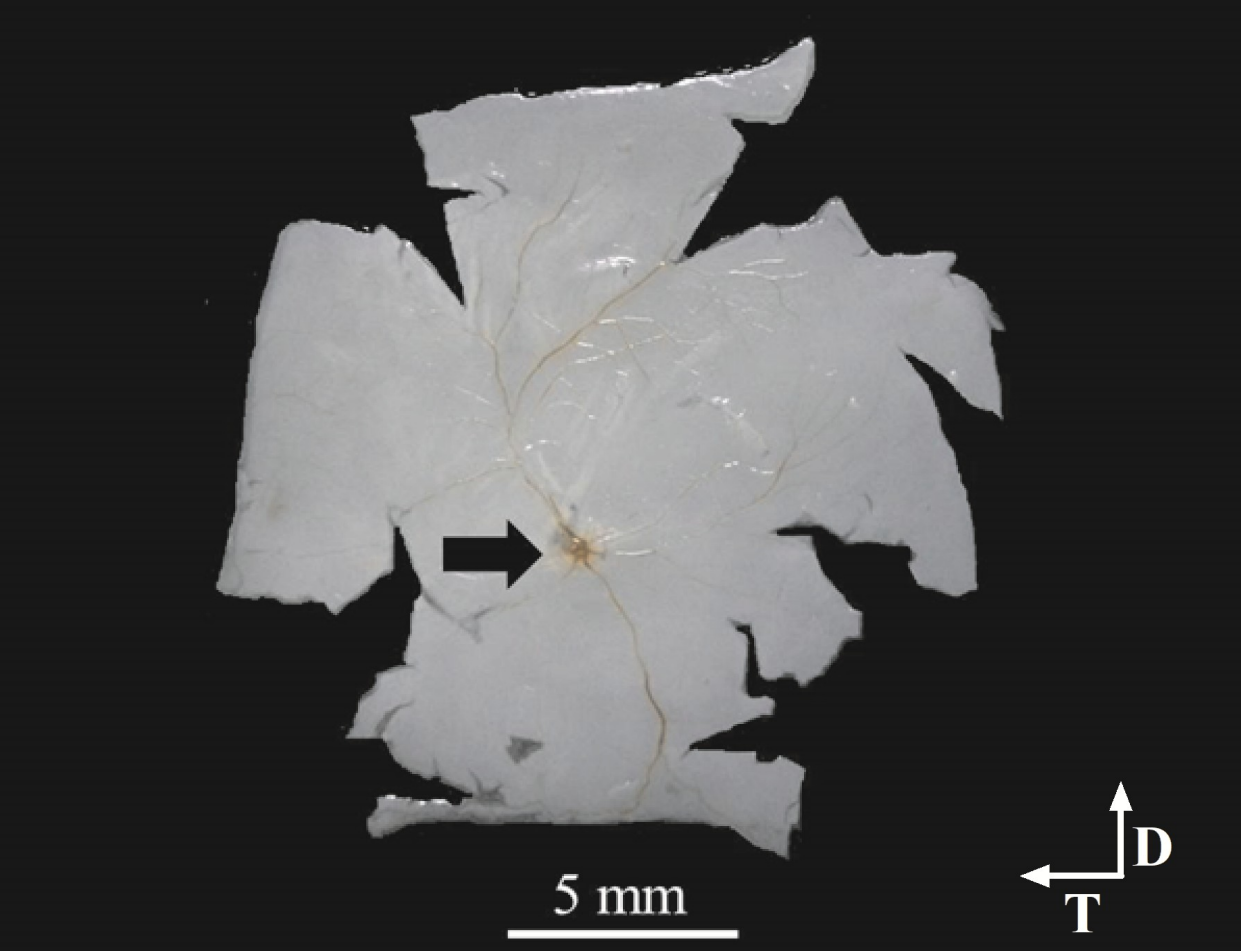
Wholemount retina of peccary. The retina was flattened on gelatin-coated slides right after the histological dissection. Blood vessels can be seen converging to the optic disc to where the arrow is pointed.

The peccary’s retina had a typical vascular pattern called holangiotic as early described [39], the optic disc (OD) has an oval appearance. It is located just below the center of the retina and temporally displaced (Fig 1). The retinal area comprised 837.8 ± 56.5 mm^2^ (N = 6) before the histological procedure and 828.8 ± 52.3 mm^2^ after the histological procedure. The shrinkage due to histological procedures was estimated and ranged from 0.40% to 1.87%, a compilation of retinal area measurements performed before and after histology is showed on Table 1. The shrunken area was restricted to the periphery. Thus, ganglion cell counting was completed with no corrections for shrinkage.

**Table 1:** Retinal area, shrinkage and total ganglion cells number.

Retina	Eye	Area (mm^2^)		Shrinkage	Total ganglion cells
		before	After		
Animal 01	Right	752	749	0.40%	994,818
Animal 01	Left	791	786	0.63%	907,666
Animal 02	Right	854	838	1.87%	889,134
Animal 02	Left	849	843	0.71%	1,154,780
Animal 03	Right	906	890	1.77%	1,055,662
Animal 03	Left	875	867	0.91%	1,174,927
Mean		837.8	828.8	1.05%	1,029,497
Std. Deviation		56.5	52.3	0.62%	121,060

**Fig 2:**
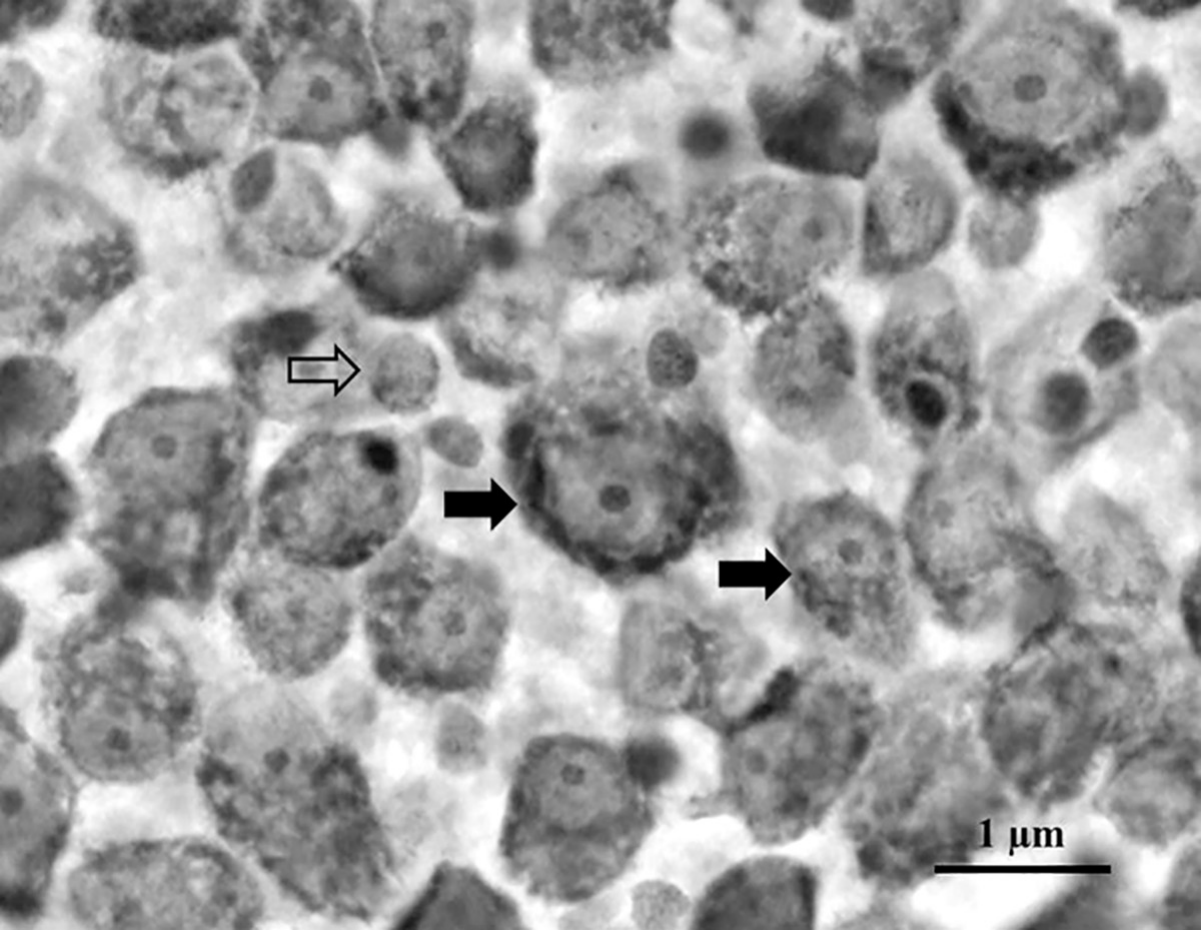
Nissl-stained of the whole-mounted retina. Ganglion cells are indicated by filled arrows and displaced amacrine cells by the empty arrow. Ganglion cells have a large soma with pale nucleus and nucleolus with abundant Nissl substance. Displaced amacrine cells have small soma and cytoplasm containing less Nissl substance. Scale bar = 1μm.

We used morphological criteria to differentiate ganglion cells from neuroglial cells and displaced amacrine cells according to Hughes [41] The same criteria was used in other studies for artiodactyls species [32, 42, 43] and other species [17, 40, 44]. Briefly, ganglion cells have a large soma with an oval pale nucleus and a prominent nucleolus with abundant Nissl substance visible in the cytoplasm. On the other hand, displaced amacrine cells have small soma and cytoplasm containing less Nissl (Fig 2).

### Total number and topography of ganglion cells

**Fig 3:**
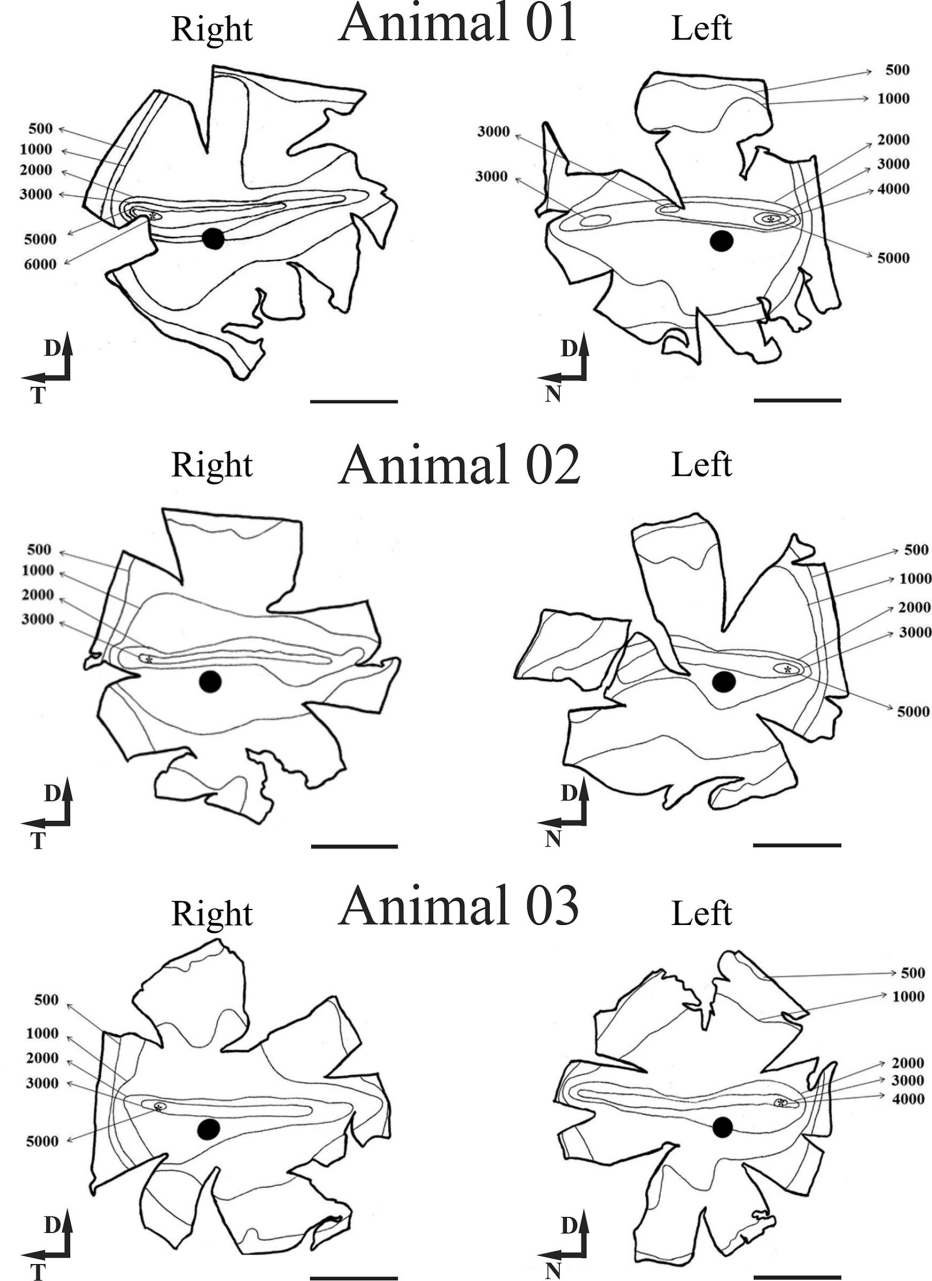
Ganglion cell isodensity maps for peccary’s retina. The contours correspond to the isodensity lines. The visual streak is visible by the horizontal elongation of the contours in the centro-dorsal retina. (*) Peak density local, (●) optic disc. Scale bar = 5 mm.

The total of the ganglion and displaced amacrine cells was estimated by the integration of isodensity contours from the isodensity maps (Fig 3). The total number of ganglion cells for all retinas was 1,029,498 (±121,060) (Table 1). Table 2 shows the descriptive statistic for the density of ganglion cells; the mean density for all retinas analyzed was 2254 GC/mm^2^ (± 346.7). The animal 03 showed the lower values, around 1,990 GC/mm^2^ for both retinas. The density peak also varied strongly, the highest value was 9,900 GC/mm^2^, and the lower value was 5,100 GC/mm^2^, with peak mean at 6,767 GC/mm^2^ (±1,914).

**Table 2:** Descriptive statistics of the ganglion cell density for all six retinas.

		Animal 01		Animal 02		Animal 03	
		Right eye	Left eye	Right eye	Left eye	Right eye	Left eye
	Number of counting sites	331	318	372	318	433	437
Ganglion cells density (GC/mm^2^)	Mean density	2634	2027	2276	2704	1890	1990
	Std. Deviation	2154	1876	1436	1536	1296	1371
	Std. Error of Mean	118.4	105.2	74.46	86.15	62.27	65.61
	Minimum	75	100	75	200	250	37
	25% Percentile	800	600	700	900	700	700
	Median	1700	1100	2700	3150	1500	1600
	75% Percentile	4200	3200	3400	3800	3100	3300
	Maximum (Peak of density)	9900	8200	5700	6500	5100	5200

**Fig 4:**
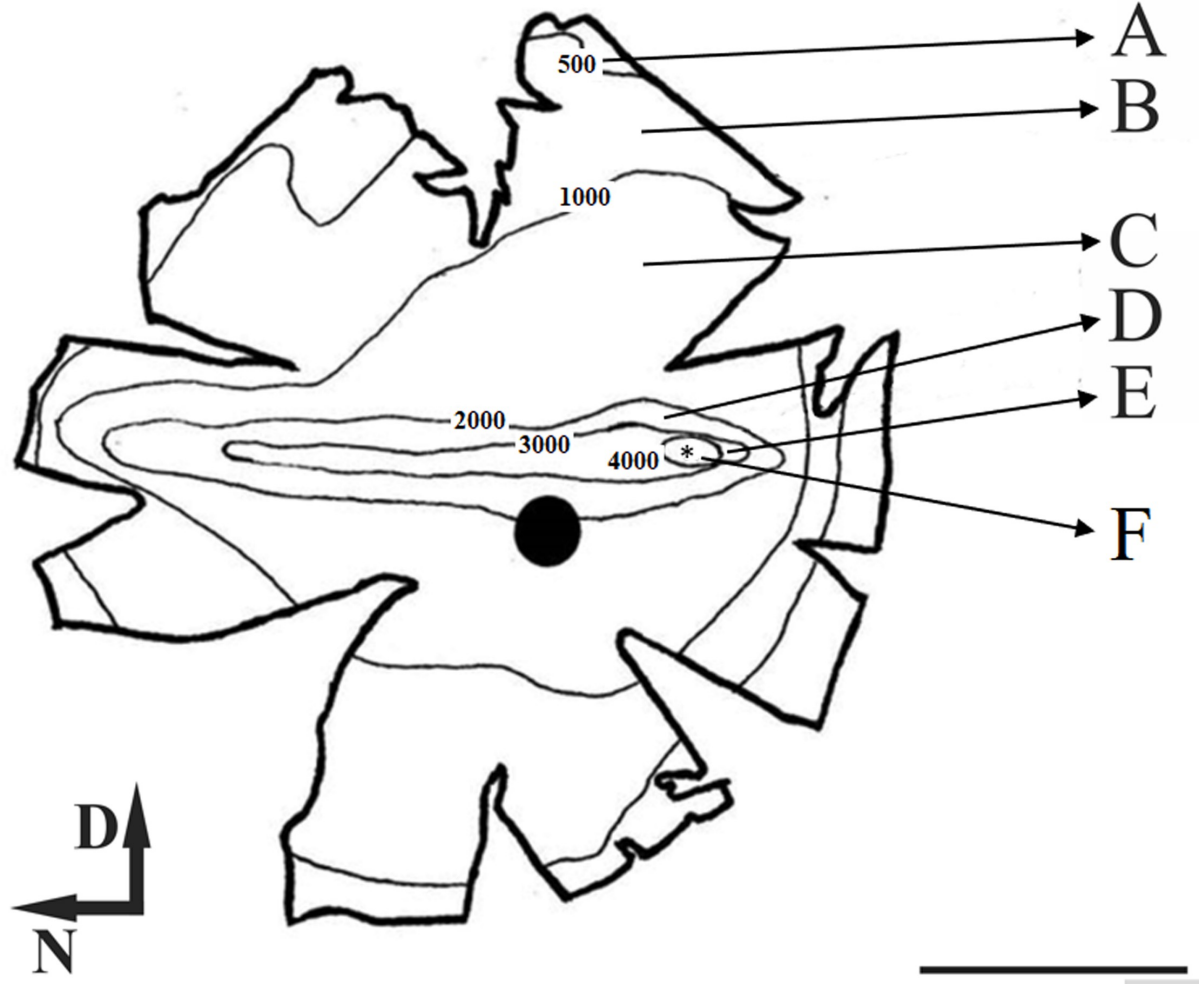
Ganglion cell mean isodensity map of peccary’s retina. The contours correspond to the isodensity lines. The visual streak is visible by the horizontal elongation of the contours in the centro-dorsal retina. Each letter represents a different region of cellular density, (A) corresponded to the area between the wholemount border and the 500 GC/mm^2^ contour. The (B) area between 500 and 1000 GC /mm^2^; (C) area between 1000 and 2000 GC/mm^2^; (D) region between 2000 and 3000 CG/mm^2^; (E) region between 3000 and 4000 GC/mm^2^, (F) region between 4000 GC/mm^2^ and Peak density (*), (●) optic nerve. Scale bar = 5 mm.

**Fig 5:**
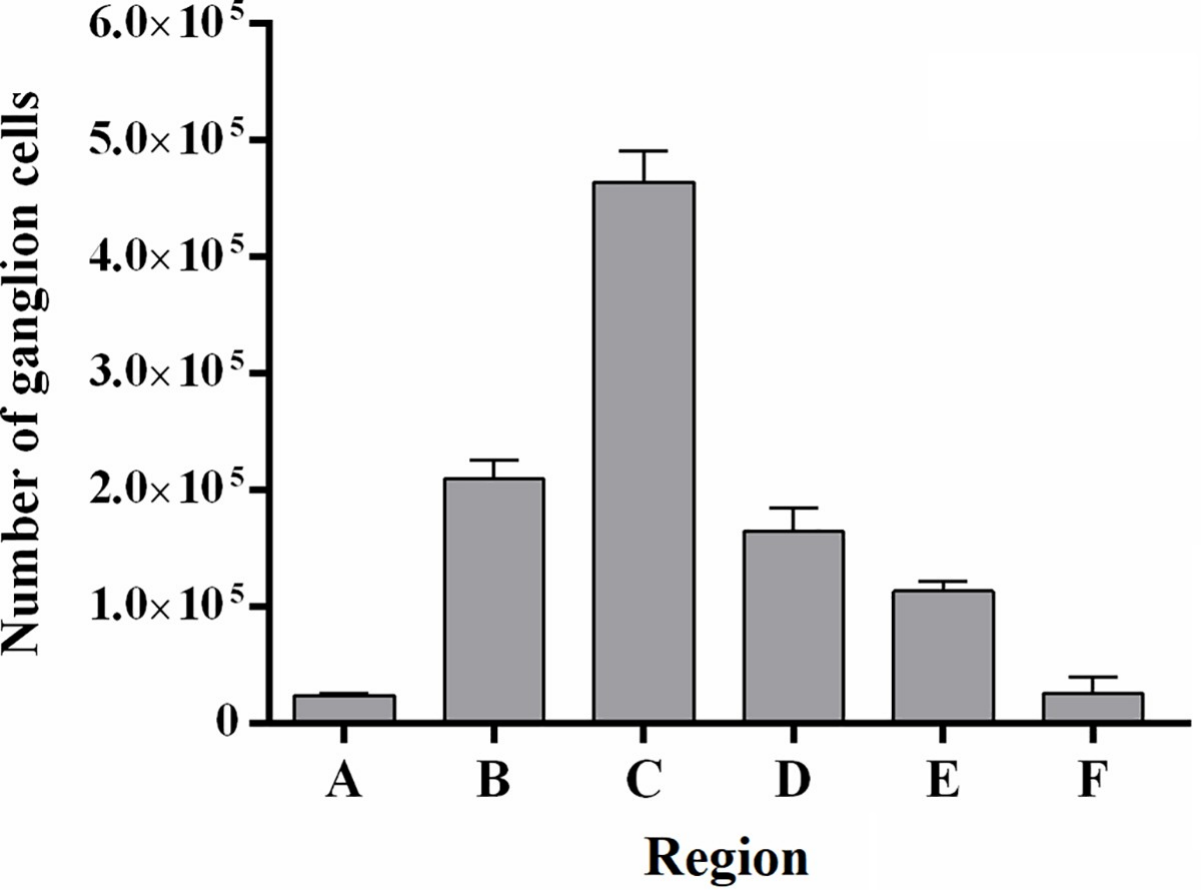
Number of ganglion cells by area. The A region corresponded to the area between the whole mount border and the 500 GC/mm^2^ contour. The B region was the area between 500 and 1000 GC/mm^2^; C region was the area between 1000 and 2000 GC/mm^2^; D region was the area between 2000 and 3000 GC/mm^2^; E region was the area between 3000 and 4000 GC/mm^2^; F was the area between 4000 and 5000 GC/mm^2^ and G region was the area on the peak density.

When we analyzed the ganglion cell isodensity maps (Fig 3), it can be seen that the lower cellular density occurred at the retinal periphery and was around 500 GC/mm^2^. The density increases concentrically towards the visual streak with density contours varying from around 2000 to 4000 GC/mm^2^. Besides, in visual streak, there was a temporarily dislocated circular region with densities between 4000 and 5000 GC/mm^2^, inside this area it´s located the peak density (*), this area is called *area temporalis* [36]. Fig 4 shows the “average” isodensity map for all retinas used in this study. For the ‘‘average” map, the isodensity contours were drawn from mean density values of six retinas and plotted on the map of Animal 03 (left retina). Here we analyzed the number of ganglion cells by region. Each region was defined for lines isodensity contours. Thus, region ***A*** corresponded to the area between the wholemount border and the 500 GC/mm^2^ contour. The ***B*** region is the area between 500 and 1000 GC /mm^2^; ***C*** region was the area between 1000 and 2000 GC/mm^2^; ***D*** region was the area between 2000 and 3000 GC/mm^2^; ***E*** region was the area between 3000 and 4000 GC/mm^2^; ***F*** region was the area between 4000 GC/mm^2^ and density peak area represent by (*). In Fig 5 we presented a column graphic compared to the number of ganglion cells by region identify in the “average” isodensity map showed in Fig 4. Here we do not consider the density peak for this comparison. We observed that the region with most ganglion cells was ***C*,** followed for the ***B*** and ***D*** region.

**Fig 6:**
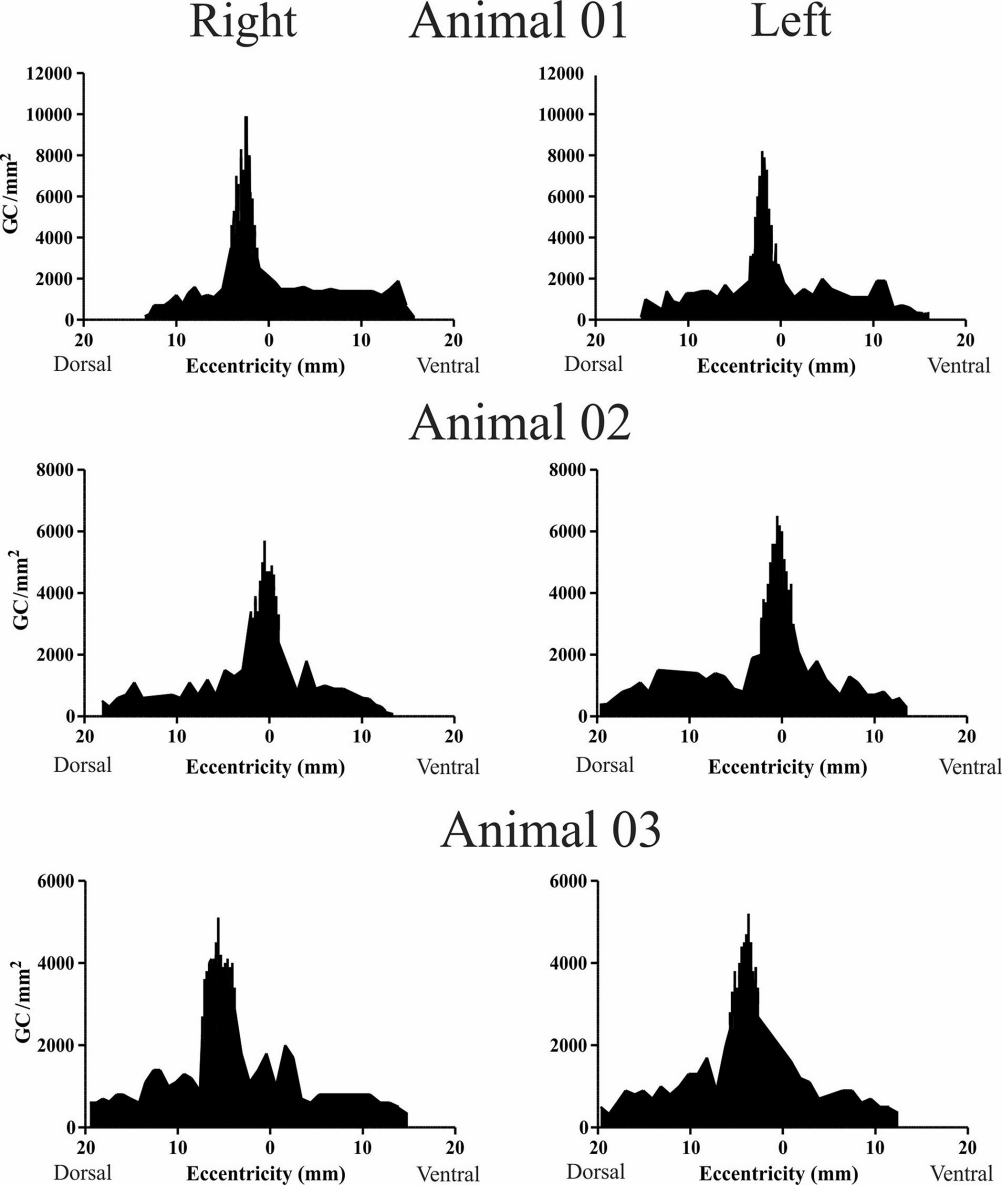
Ganglion cell density (cells/mm^2^) along the vertical axis (dorso-ventral), perpendicular to the visual streak. Values in the x-axis indicate the distance in millimeters (mm) relative to the optic nerve (zero).

**Fig 7:**
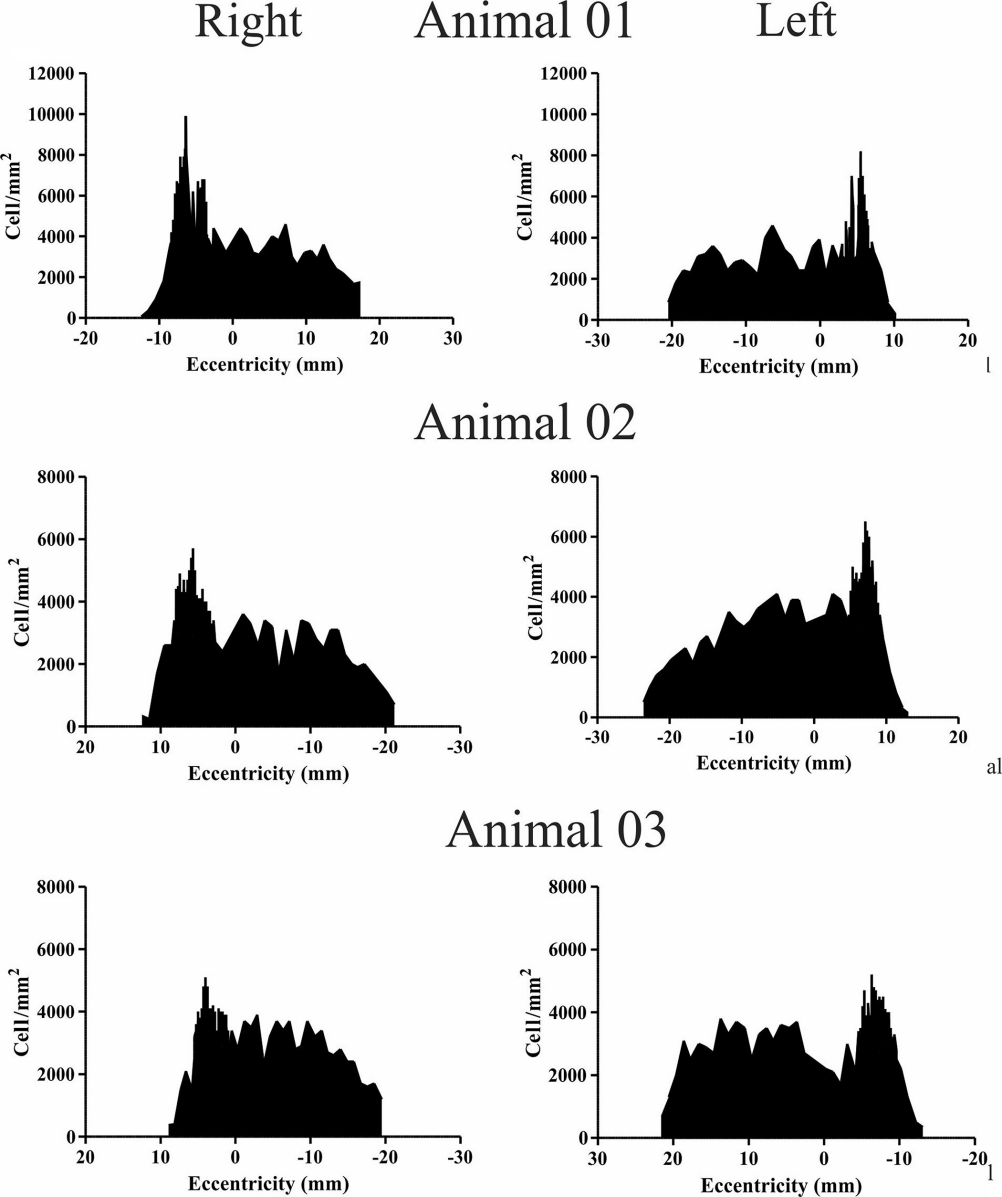
Ganglion cell density (cells/mm^2^) along the horizontal axis (naso-temporal), along the visual streak. Values in the x axis indicate the distance in millimeters (mm) relative to the optic nerve (zero).

The Figs 6 and 7 show the ganglion cells density profile in the dorsal-ventral and temporal-nasal axes, respectively. For the dorsal-ventral axis (Fig 6) the peak density was temporarily located at a mean distance of 3.13 mm (± 0.38) from the dorsal region to the optic nerve. The peak density varied among retinas with values ranging from 5100 to 9900 GC/mm^2^ (Table 2). From the optic nerve, there is an abrupt decrease in cell density to values close to 1000 GC/mm^2^ in the dorsal and ventral. Similarly, in the dorsal and ventral periphery, especially between the retinal border and first-line contour, the density falls to about 500 GC/mm^2^.

For the temporal-nasal axis, along the visual streak (Fig 7), the density was around 4000 GC/mm^2^ from nasal to central direction. The peak density was temporarily located at approximately 6.77 mm (± 0.60) from the optic nerve.

### Displaced amacrine cells

**Fig 8:**
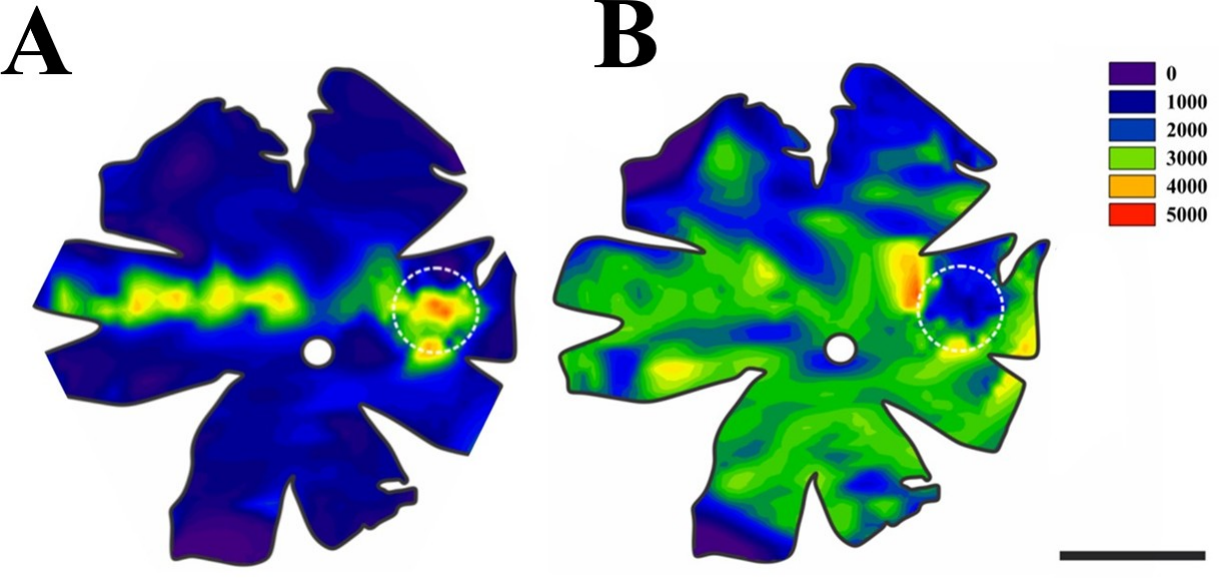
Mean cell density in the peccary’s retina. The color scale on the right indicates density variation. **(A)** Ganglion cell density, the visual streak can be easily seen as a horizontal narrowly band in the naso-temporal axis. **(B)** Displaced amacrine cell density was approximately homogeneous overall retina surface. Interestingly, in the *area temporalis*, there was an intense decrease in the density of displaced amacrine cells. For both retinas, the *area temporalis* and the optic nerve is indicated by the white dotted circle and white disc, respectively. Scale bar = 5 mm.

The topographical distribution of displaced amacrine cells (DAC) differs significantly from the ganglion cells distribution in two critical aspects: First, there was no spatial arrangement such as a visual streak; second, there was a significant decrement of displaced amacrine cells in the *area temporalis* (Fig 8). The distribution of displaced amacrine cells is homogeneous in the majority of the retina (Fig 8B) with a density of around 3000 DAC/mm^2^, and in the *area temporalis* region (white stippled circle), there was a considerable decrease in the density of displaced amacrine cells.

**Fig 9:**
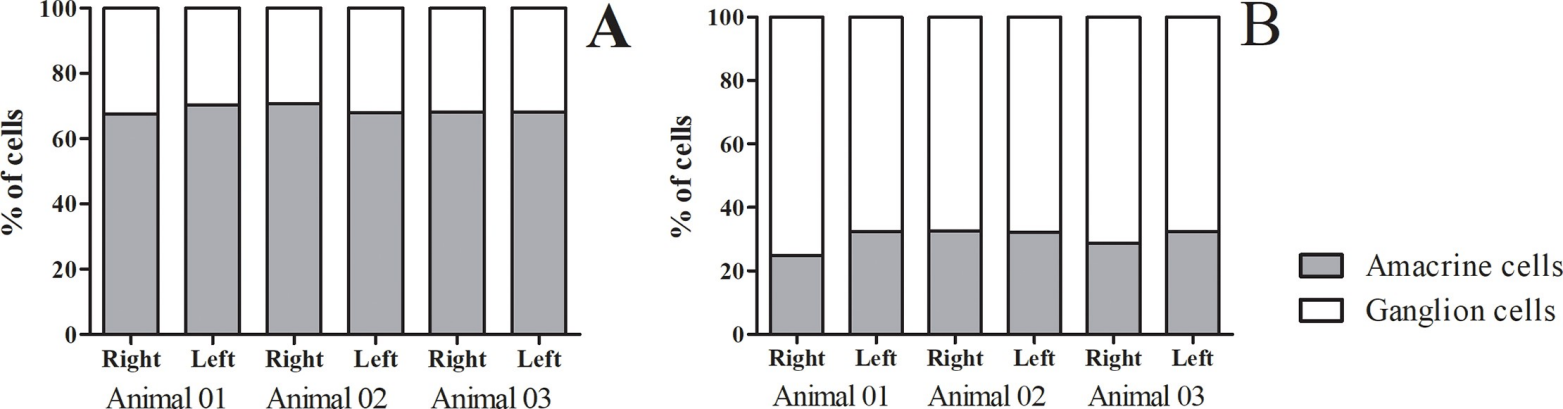
Stacked column graph of ganglion and amacrine cells for all six retinas studied. **(A)** Stacked column graph of percentage of amacrine and ganglion cells in the whole retina, except *area temporalis*. Each column represents 100% of cells in ganglion cell layer per retina, the percentage of amacrine cells is represented by the gray region and the percentage of ganglion cells is represented by white. It’s noticeable that amacrine cells are in higher percentage than ganglion cells, the percentage of amacrine cells is around 70% and 30% for ganglion cells. **(B)** When just the *area temporalis* is considered, there was an inversion in the proportion of amacrine and ganglion cells. Ganglion cells were in a higher percentage than amacrine cells.

From the neurons in the ganglion cell layer the percentage of amacrine cells was always larger (reaching up to 70%) than the percentage of ganglion cells except for the *area temporalis* (Fig 9A). Notably, in the *area temporalis*, there was an inversion of the percentage of amacrine and ganglion cells where the amacrine cells decreased to 30.52% ± 3.91 on average against 69.48% ±3.09 of ganglion cells (Fig 9B).

## Discussion

In the present study, we have investigated the density distribution of ganglion and displaced amacrine cells using six retinas from the collared peccary, a diurnal mammal found throughout all Amazon Rainforest. The ganglion cell layer showed three characteristic regions with high cellular density: visual streak; *area temporalis* and a dorsotemporal extension named anakatabatic area, the later also found in other animals such as giraffe [32]. We located the visual streak above the optic nerve and the *area temporalis* superposed on the visual streak and the dorso temporal extension forming a dorsal arch of increased number of ganglion cells.

These results suggested that despite the difference in mean density values as well as peak density values, collared peccary and other species of the order Artiodactyla seem to share essential characteristics like retinal organization in ganglion cells layer. For example, in peccary, the mean cell density of ganglion cells in all retinas analyzed was 2253.5 GC/mm^2^, this value is higher than was reported for the wild pig (*Sus scrofa*), whose mean density was about 1133 GC/mm^2^ [31]. On the other hand, if we consider just the peak density value for collared peccary, the average was 6767±1.94 GC/mm^2^, which is very close to what was found for wild pig’s retina, that was 5735 ± 1066 GC/mm^2^ [31]. Besides, the density peaks in both animals are displaced temporally relative to the optic nerve inside the visual streak. When our results for the collared peccary are compared to the retina topography analysis carried out in other species from the Artiodactyla order, some similarities are also noticed. For the goat, Gonzales-Soriano et al., reported a peak density of 3592 GC/mm^2^ restricted to a circular area temporal to the optic disc, besides the existence of a visual streak [34]. In another study, this time in adult sheep retinas, the authors also found a visual streak formed by ganglion cells and *area temporalis* with a peak density of 18,000 GC/mm^2^ [35]. These visual specializations (visual streak and *area temporalis*) were described in many artiodactyls such as giraffes [32], goats [41], cattle, pigs, and sheep [32, 33], animals that inhabit a variety of environments, from the savannah to dense rainforests. Whereas the dorsal extension was found in artiodactyls such as sheep [35], giraffes [32], and the Nubian ibex [42].

Coimbra et al., [32] argued based on the theory of phylogenetic ancestry of Stone [45] that, as the horizontal streak and area temporalis are frequent in retinas of artiodactyls, these specializations are most likely plesiomorphic characters inherited from a common ancestor. Thus, regardless of habitat or lifestyle, the visual streak and area temporalis are conserved traits in the retina of the order [26, 31, 32, 34, 46]. Based on Hughes [47], Coimbra et al., [32] also claim that the joint presence of these two conspicuous retinal characteristics certainly indicates their relation with important behavioral marks like foraging and avoiding predators. The dorsotemporal extension forms a dorsal arch of ganglion cells increase Some authors reported that this specialization is closely related with demands required by the inferior visual field such as spotting predators, orientation during foraging or ambulation [32]. It was also found in other artiodactyl living in mountains and valleys [42], which points to a shared ecological niche.

Undoubtedly, independent of the topographical arrangement of retinal cells, the ubiquitous presence of the visual streak, dorsotemporal extension and area temporalis provide a good adaptation despite the variety of natural environments inhabited for these animals. In this respect, we can conclude that the distribution of ganglion cells in the retina of the collared peccary is not different from the distribution of ganglion cells observed in the retinas of other diurnal artiodactyls. Interestingly, the collared peccary is found in a wide range of environments such as desertic regions and tropical forests, suggesting again that the topographical map described for ganglion cells serves well for different habitats being important for foraging and avoiding predators.

### Displaced amacrine cells

The displaced amacrine cells have been reported in other species but with little emphasis on their topographical distribution [48–54]. The topography of amacrine cells in the collared peccary retina differs from ganglion cell distribution in some critical aspects related to the retina specializations, visual steak, dorsotemporal extension and *area temporalis*.

The topography of the amacrine cells displaced in the peccary's retina is almost homogeneous throughout its extension and does not form a retinal specialization. Except in the area centralis, the proportion of displaced amacrine cells in the ganglion cell layer of the peccary retina is higher than the proportion of ganglion cells. Likewise, displaced amacrine cells are absent from the central area in the retina of the teleost thraira [52]. In addition, studies of a variety of primate retinas have shown that the proportion of displaced amacrine cells in the peripheral retina is significantly higher than that in central retina [14, 55, 56].

In the howler monkey (*alouatta caraya*), a diurnal primate, the proportion of amacrine cells versus ganglion cells is like that found in the collared peccary. In the alouatta on the periphery of the retina, amacrine cells represent about 67% to 84% of the total cells in the ganglion cell layer. In contrast, in the central retina (fovea) there is an inversion in the proportion of amacrine and ganglion cells, and amacrine cells represent about 6% of the cells in that region [14]. In aotus, a nocturnal primate, the proportion of amacrine and ganglion cells is similar in most of the retina, except for the central region where displaced amacrine cells also decrease their percentage concerning ganglion cells [56].

The peculiarities and similarities found in the peccary’ retina concerning other species, represent essential features of the order Artiodactyla to which the *P*. *tajacu* is inserted. The presence of retinal specializations in the peccary’s retina, such as the visual streak, dorsotemporal extension and the *area temporalis*, which are directly related to its evolutionary history and ecology of the species, allow initiating morphophysiological comparisons of the retina of the collared peccary with that of other animal species.

## Supporting information

S1 Dataset Coordinates, density and percentage of ganglion and amacrine cells by Animal 01 Right. (XLSX)

S2 Dataset Coordinates, density and percentage of ganglion and amacrine cells by Animal 01 Left. (XLSX)

S3 Dataset Coordinates, density and percentage of ganglion and amacrine cells by Animal 02 Right. (XLSX)

S4 Dataset Coordinates, density and percentage of ganglion and amacrine cells by Animal 02 Left. (XLSX)

S5 Dataset Coordinates, density and percentage of ganglion and amacrine cells by Animal 03 Right. (XLSX)

S6 Dataset Coordinates, density and percentage of ganglion and amacrine cells by Animal 03 Left. (XLSX)
